# Efficacy and safety of a fixed dose artesunate-sulphamethoxypyrazine-pyrimethamine compared to artemether-lumefantrine for the treatment of uncomplicated *falciparum *malaria across Africa: a randomized multi-centre trial

**DOI:** 10.1186/1475-2875-8-63

**Published:** 2009-04-14

**Authors:** Issaka Sagara, Stephen Rulisa, Wilfred Mbacham, Ishag Adam, Kourane Sissoko, Hamma Maiga, Oumar B Traore, Niawanlou Dara, Yahia T Dicko, Alassane Dicko, Abdoulaye Djimdé, F Herwig Jansen, Ogobara K Doumbo

**Affiliations:** 1 Malaria Research and Training Center, Department of Epidemiology of Parasitic Diseases, Faculty of Medicine, Pharmacy and Odonto-Stomatology, University of Bamako, Bamako, Mali; 2 Kigali Central University Hospital, Kigali Rwanda; 3 The Laboratory for Public Health Biotechnology, The Biotechnology Centre, University of Yaounde I, Yaounde, Cameroon; 4 University of Khartoum, Khartoum, Sudan; 5 Dafra Pharma NV/SA, Slachthuisstraat 30/7, 2300 Turnhout, Belgium

## Abstract

**Background:**

The efficacy of artemisinin-based combination therapy has already been demonstrated in a number of studies all over the world, and some of them can be regarded as comparably effective. Ease of administration of anti-malarial treatments with shorter courses and fewer tablets may be key determinant of compliance.

**Methods:**

Patients with uncomplicated falciparum malaria and over six months of age were recruited in Cameroon, Mali, Rwanda and Sudan. 1,384 patients were randomly assigned to receive artesunate-sulphamethoxypyrazine-pyrimethamine (AS-SMP) three-day (once daily for 3 days) regimen (N = 476) or AS-SMP 24-hour (0 h, 12 h, 24 h) regimen (N = 458) or artemether-lumefantrine (AL), the regular 6 doses regimen (N = 450). The primary objective was to demonstrate non-inferiority (using a margin of -6%) of AS-SMP 24 hours or AS-SMP three days versus AL on the PCR-corrected 28-day cure rate.

**Results:**

The PCR corrected 28-day cure rate on the intention to treat (ITT) analysis population were: 96.0%(457/476) in the AS-SMP three-day group, 93.7%(429/458) in the AS-SMP 24-hour group and 92.0%(414/450) in the AL group. Likewise, the cure rates on the PP analysis population were high: 99.3%(432/437) in the AS-SMP three-day group, 99.5%(416/419) in the AS-SMP 24-hour group and 99.7(391/394)% in the AL group. Most common drug-related adverse events were gastrointestinal symptoms (such as vomiting and diarrhea) which were slightly higher in the AS-SMP 24-hour group.

**Conclusion:**

AS-SMP three days or AS-SMP 24 hours are safe, are as efficacious as AL, and are well tolerated.

**Trial registration:**

NCT00484900 .

## Background

Over the last few years artemisinin-based combination therapy (ACT) is widely accepted as an appropriate treatment for malaria. This disease remains an important killer, particularly in childhood, in sub-Saharan Africa [1-3]. ACT offers new therapeutic possibilities and the World Health Organization (WHO) has recommended a series of such combinations with several studies in progress [4].

The efficacy of ACT has already been demonstrated in a number of studies all over the world [5-7], and some of them can be regarded as comparably effective, when using adequate clinical and parasitological responses as a measure of final outcome [8]. One of the conclusions of this analysis states that secondary factors, such as side effects, ease of administration, cost, duration of the treatment, become important, when selecting the appropriate treatment. Ease of administration of anti-malarial treatments is a key determinant of compliance and, therefore, efficacy, with shorter courses and fewer tablets being preferred over the current minimum of three days and multiple tablets a day for most forms of ACT. These secondary factors must be considered when selecting an anti-malarial treatment, since they can greatly influence the final outcome. A fixed-dose artemisinin-based combination (FDC) treatment would be able to improve compliance of the treatment and reduce the cost of malarial treatment in endemic countries in Africa.

In a recent communication, WHO experts announced that the ideal anti-malarial drug should have an efficacy of at least 95% as measured over 28 days of follow-up. They recommend that re-infection in that period should be minimal and that, ideally, the treatment should be restricted to a few pills administered as a single dose and should have a short treatment duration [9]. To try and improve the existing ACT, artesunate (AS) was combined with sulphamethoxypyrazine-pyrimethamine (SMP) in a co-blister. This combination of AS-SMP (Co-Arinate^®^), taken once daily (two tablets simultaneously) over three days, was studied in Mali [10], Ivory Coast (Dafra Pharma file), Rwanda [11], and is available as a prescription drug in numerous African countries. Based on this experience, a fixed-dose combination (Co-Arinate FDC^®^), was subsequently developed. Since preliminary experiments with these tablets indicated that the dosing interval could be reduced to 12 hours enabling a 24-hour therapy [12,13], it was of interest to evaluate in a larger population whether this dosage regimen is equally effective compared with the same treatment given over 48 hours (dose interval of 24 hours). It was decided to use the WHO standard essential drug Coartem^® ^(AL FDC), available as a six-dose regimen, as standard therapy for assessing AS-SMP FDC. The primary objective was to demonstrate the non-inferiority of AS-SMP 24 hours or AS-SMP three days versus AL on the PCR-corrected 28-day cure rate.

## Methods

### Study sites

This multi-centre study, which took place in four African countries: Cameroon, Mali, Rwanda and Sudan. In Cameroon, the study took place in the capital city, Yaoundé. The study site was the Cameroon Baptist Convention clinic in the peri-urban district of Biyem-Assi. Transmission in Yaoundé occurs in two peak periods at the start of the rainy season in March/April, and in October/November as the rains cease. Chloroquine resistance is above 45%, amodiaquine resistance is about 10% and resistance to sulphadoxine/pyrimethamine (SP) is higher than 10% in Yaoundé or other parts of Cameroon [14,15].

In Mali, the study took place at Bancoumana health center and surrounding villages (Kolle and Samako). The study area is located at about 60 km south-west of Bamako. The main population activity is farming. Malaria is hyperendemic and the transmission is mainly seasonal from June to December. *Plasmodium falciparum *is the predominant plasmodium species, accounting for more than 95% of malaria cases. Chloroquine resistance is above 25% [16,17] and resistance to SP is around 5% in a part of this area.

In Rwanda, the study took place in Rwamagana and Muhimai. The study area is located at about 50 km east of Kigali. The main population activity is farming. Malaria is hyperendemic. *Plasmodium falciparum *is the predominant plasmodium species, accounting for more than 95% of malaria cases. Resistance to SP exceeded 25% [18].

In Sudan the study took place at Alhara Alola health center in New Hlafa, eastern Sudan. This area is located at 500 km from Khartoum in the middle of the second largest irrigated agricultural scheme in Sudan. Cotton and wheat are the main crops cultivated during the winter season. The area is characterized by a high level *of P. falciparum *resistance to chloroquine (>70%) and the reported SP resistance in Sudan is ranging from 5% to 30% [19,20].

### Patients

This study recruited patients presenting at the health center with typical symptoms of malaria and who had a blood smear positive for *P. falciparum*. Inclusion criteria were: above six months of age, weight above5 kg, infection with *P. falciparum *(asexual stages 2,000–200,000/μL) at screening, fever (axillary temperature ≥37.5°C or a history of fever in the preceding 24 hours), resident of study site, able to take oral treatment. Exclusion criteria were: symptoms or signs of severe malaria [21], serious underlying diseases or any other illness that required a treatment non-compatible with the study, allergy to study drugs, use of any component of the study drugs within the last 28 days prior enrollment, and pregnancy (detected clinically, or with beta human chorionic gonadotrophin test (HCG)).

### Study design

This was a prospective in vivo efficacy study. It was a randomized open label trial comparing fixed dose AS-SMP 24-hour or three-day regimen to AL as the standard reference treatment. The laboratory personnel assessing the parasitaemia was kept blinded. The study was carried out according to current WHO 2003 Protocol [22].

### Study procedures and treatment

Enrolled patients were randomly assigned a fixed dose of AS-SMP (Co-Arinate FDC^®^, Dafra Pharma, Belgium), as a 24-hour or three-day regimen, or AL (Coartem^®^, Novartis). The randomization code was computer-generated by a third party not involved in patients' outcome assessment. It was a block randomization procedure stratified by country. Study codes were sealed in individual envelopes and securely stored. Randomized patients were assigned a study number in numerical sequence by the investigators. All drugs were manufactured according to Good Manufacturing Practice (GMP). AL tablets were a fixed combination, each containing 20 mg of artemether and 120 mg of lumefantrine. AL was administrated according to body weight (5–14 kg: one tablet; 15–24 kg: two tablets; 25–34 kg: three tablets; ≥35 kg: four tablets) as six consecutive doses: The first dose at diagnosis and the second dose eight hours later on Day 0, and then two doses at 12 hourly intervals for the subsequent two days.

AS-SMP FDC is available as adult, junior and baby treatment packs. The adult tablet contains 200 mg artesunate, 500 mg of sulphamethoxypyrazine and 25 mg of pyrimethamine, while each junior tablet contains 100 mg of artesunate, 250 mg of sulphamethoxypyrazine and 12.5 mg of pyrimethamine, and each baby tablet contains 50 mg of artesunate, 125 mg of sulphamethoxypyrazine and 6.25 mg of pyrimethamine. The dosage for this study was determined by weight, with adults weighing 40 – 79 kg and >79 kg receiving one tablet and one and a half tablet of the adult preparation, respectively. One tablet of junior preparation was given to individuals weighing between 20 and 40 kg and the baby preparation was given at a dose of one and a half tablets, one tablet and a half tablet for children weighing between 13 and 20 kg; between eight and 13 kg, and between five and eight kg, respectively.

Patients allocated to the three-day treatment with AS-SMP FDC were given their treatment three times, at an interval of 24 hours (0 h – 24 h – 48 h). Patients allocated to the 24-hour treatment with AS-SMP FDC were given tablets for three consecutive intakes at an interval of 12 hours (0 h – 12 h – 24 h).

All study drug doses were administrated at the heath center by the study team. A full drug dose was re-administrated if the patients either spat out or vomited the study drugs within 30 minutes. Half the drug dose was re-administrated if the patient vomited the study drugs between 30 minutes to one hour. If the patient rejected again, he/she received another anti-malarial drug in conformity with the National Malaria Control Programme of his/her respective country, and was then excluded from the study.

Follow-up examinations were made on days 0, 1, 2, 3, 7, 14, 21 and 28 or at any time if patient felt unwell. Blood from a finger prick was obtained to make a thick smear and a filter paper dot during each day of follow-up. The patients or guardians were asked about drug consumption since the last clinic visit. Individuals for whom treatment failed were treated according to National Malaria Control Programme. Giemsa-stained thick smears were read by an experienced microscopist blinded to study arm. Parasitaemia was quantified by a standard approximation method (40 × number of parasites per 200 leucocytes on the thick film). Slide quality control was done by masked re-reading of 10% of slides, selected randomly. Haematological tests (Complete Blood Count) and biochemistry analyses (concentrations of alanine aminotransferase, bilirubin and creatinine) were done at baseline and at day 7 and then on day 28. Patients at Cameroon site and few patients at Sudan site did the blood test on day 14 instead of day 7. The tests were done any time for any subject if clinically recommended or in case of significant abnormality in tests during scheduled lab visits. Venous whole blood collected in anticoagulant tube was used for haematological tests, while venous blood collected in serum separator tube was used for biochemistry analyses. Less than 10 ml of blood was needed for these tests.

For participants with recurrent parasitaemia after day 7, paired polymerase chain reaction (PCR) blots (from day 0 and the day of parasitaemia recurrence) were analysed for parasite merozoite surface protein 1 and 2 genes (*msp1 *and *msp2*) and microsatellite (CA1), to distinguish between re-infection and recrudescence as described previously [23]. A comparison was made with the day 0 and failure day alleles of *msp1 *and *msp2 *and the microsatellite CA1 gene loci.

Possible outcomes were: (i) recrudescence, if the alleles of the pre- and post-treatment samples were the same for *msp-1, msp-2 *and CA1; (ii) re-infection, if the alleles of the pre- and post-treatment samples are distinct for any one of these loci; (iii) mixed recrudescence and re-infection, if similar alleles are found in the pre- and post-treatment samples for all the markers as mentioned above, but with additional distinct alleles identified; (iv) indeterminate, if either or both the pre- and post-treatment samples could not be amplified. Mixed recrudescent and re-infection cases were computed as recrudescent.

### Outcome measures

The classification of the therapeutic outcome was done according to the current 28-day WHO protocol [22]. The primary endpoint was the 28-day cure rates and was defined as proportion of patients with PCR-corrected adequate clinical and parasitological response (ACPR) after 28 days of follow-up. Secondary endpoints were early treatment failure (ETF), late clinical failure (LCF), late parasitological failure (LPR), adverse events (clinical and laboratory abnormalities), anaemia (haemoglobin value<10 g/dl), clearance rate of fever and parasitaemia, and gametocyte carriage. Parasite and fever clearances were assessed on days 1, 2, and 3. The gametocyte carriage was assessed on days 0, 3, 7, 14, 21, and 28. Adverse event was defined as a sign, symptom, or abnormal laboratory value not present on day 0, but which occurred during follow-up, or was present on day 0 but became worse during follow-up. Serious adverse events were defined according to ICH (International Conference on Harmonization) guidelines.

### Statistical analysis

The non-inferiority of the AS-SMP 24 hours or AS-SMP 3 days to AL on the 28-day PCR-corrected cure rates was assessed by constructing a one-sided, lower limit, asymptotic 97.5% confidence interval (CI) on the difference in cure rates between AS-SMP 24 hours or AS-SMP 3 days and AL. Non-inferiority was declared if the lower limit of this CI was greater than -6% (for AS-SMP 24 hours or AS-SMP three days minus AL). The non-inferiority margin of 6% was chosen from a cure rate of 94% at day 28 with AL reported in the literature [7]. Likewise, the non-inferiority of the AS-SMP 24 hours to AS-SMP 3 days on the 28-day PCR-corrected cure rates by constructing a one-sided, lower limit, asymptotic 97.5% confidence interval (CI) on the difference in cure rates between AS-SMP 24 hours and AS-SMP three days. Non-inferiority was declared if the lower limit of this CI was greater than -6% (for AS-SMP 24 hours minus AS-SMP 3 days).

nQuery Advisor 5.0 software was used for sample size calculation. On the basis of the above assumption, 900 patients (300 per treatment group) would be needed to demonstrate non-inferiority stated above with approximately 80% power. Assuming a 15% non-evaluability rate (e.g. lost to follow-up) it was planned to enroll 1035, rounded up to 1044 (348 per treatment arm). That total sample size was increased later to a total of 1,384 subjects included into the study increasing the precision of the parameters estimate (the efficacy proportion as well as of the secondary objective outcomes such as adverse events). Data from all sites were pooled and analyzed based on different populations.

The intent-to-treat (ITT) population analysis included all randomized subjects. Lost to follow-up and withdrawn subjects were considered as treatment failure cases.

The per protocol (PP) population analysis included all subjects who took study medication and made study visits up to the time of treatment failure or to the end of the study (28 days). Lost to follow-up and withdrawn subjects including protocol violation cases were excluded in this population. The primary analysis was based on the 28-day PCR corrected efficacy of both ITT and PP populations.

The baseline and safety analysis were done using ITT population. Data were double-entered, validated using Microsoft Access and analysed with STATA version 10.0 (STATA Corporation, TX, USA). Chi-square test with Fisher correction exact test was used as appropriate to compare categorical variables. Parametric or non-parametric tests were computed as appropriate to compare continues data between the three treatments arms. P value less than α = 0.05 was considered as statistically significant.

### Ethical clearance

The protocol was reviewed and approved by: the National Ethical Committee, Yaoundé (for Cameroon),, the Ethical Committee of the Faculty of Medicine, Pharmacy and Dentistry at the University of Bamako (for Mali), the National Ethical Committee, Kigali (for Rwanda), and the Ethical Review Board of the Academy of Medical Science and Technology, Faculty of Medicine (for Sudan). Each patient (or their guardian or parent) gave fully informed written consent prior the enrollment.

## Results

### Baseline characteristics

The first patient was enrolled in August 2006 and the study was completed in May 2007.

**Figure 1:**
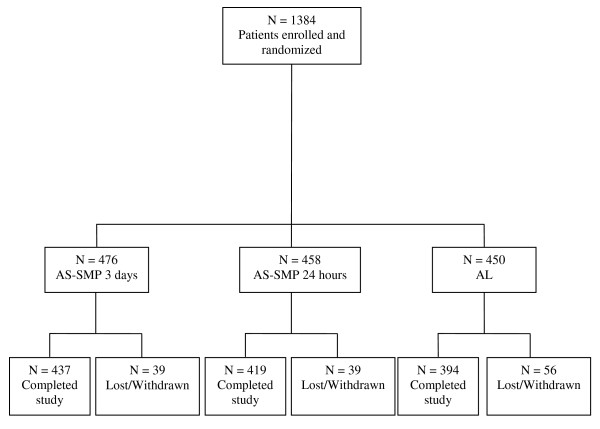
**The trial profile**.

Of the 1,384 enrolled participants (Figure 1), 476 were randomized to receive AS-SMP three days, 458 to receive AS-SMP 24 hours and 450 to receive AL. Two hundred and seventy participants were from Cameroon, 261 from Mali, 535 from Rwanda, and 318 from Sudan. Of the 1,384 participants, 1,250 completed the study (90.3%), 9.7% were lost to follow-up or were withdrawn from the study or from PP analysis (AS-SMP three days, 39/476; AS-SMP 24 hours, 39/458 AL, 56/450). Table 1 shows the baseline characteristics of all study participants. Demographic and clinical characteristics at baseline were similar between treatment groups.

**Table 1:** Baseline characteristics at enrollment

	AS-SMP 3 days (N = 476)	AS-SMP 24 hours (N = 458)	AL 3 days (N = 450)
**Age (Years)**			
Median	8.0	9.0	8.0
Min, Max	0.4, 63.5	0.6, 79.0	0.6, 60.0
**Age Group (Years)**			
< 5 – n (%)	153 (32.1)	123 (26.9)	137 (30.4)
**Gender**			
Male – n (%)	239 (50.3)	226 (49.3)	223 (49.6)
***P. falciparum *(/μl)**			
Median (IQR)	8400 (3180–24160)	8000 (3200–19520)	7600 (3120–28140)
**Gametocyte carriage **– n (%)	6 (1.3)	16 (3.5)	8(1.8)
Fever prevalence* – n (%)	332(69.7)	324(70.7)	316(70.2)

N = effective; Std Dev = standard deviation ; IQR = interquartile range

Fever = temperature ≥37.5°C at enrollment

### Efficacy

Cure rates on the ITT analysis population were: 96.0% in the AS-SMP three-day group, 93.7% in the AS-SMP 24-hour group and 92.0% in the AL group (Table 2). The lower bound of the one-sided 97.5% CI calculated around the difference between the day 28 cure rate point estimates in the AS-SMP 24-hour and AS-SMP three-day groups, AS-SMP 24-hour and AL groups, and AS-SMP three-day and AL groups were, respectively -2.3%(-5.1, 0.5), 1.7%(-1.7, 5.1) and 4.0%(0.9, 7.1) and thus greater than the pre-specified -6% non-inferiority limit. Likewise, the cure rates on the PP analysis population were high: 99.3% in the AS-SMP three-day group, 99.5% in the AS-SMP 24-hour group and 99.7% in the AL group (Table 3). The lower bound of the one-sided 97.5% CI calculated around the difference between the day 28 cure rate point estimates in the AS-SMP 24-hour and AS-SMP three-day groups, AS-SMP 24 hours and AL groups, and AS-SMP three-day and AL groups were respectively 0.2%(-0.8, 1.2), -0.2%(-1.1, 0.7) and -0.4%(-1.4, 0.6) and thus greater than the pre-specified -6% non-inferiority limit.

**Table 2:** Efficacy evaluation on Day 28 after PCR correction* Intention-To-Treat Analysis

Efficacy evaluation	AS-SMP 3 days (N = 476)	AS-SMP 24 hours (N = 458)	AL 3 days (N = 450)
Possible failure – n (%, 95%CI)	15(3.2%,1.8–5.1)	26 (5.7%,3.7–8.2)	33(7.3%,5.1–10.1)
ETF – n (%)	0	0	1(0.2%)
LCF – n (%, 95%CI)	3 (0.6%,0.1–1.8)	2 (0.4%,0.1–1.6)	0
LPF – n (%)	0	0	0
ACPR – n (%, 95%CI)	457 (96.0%,93.8–97.6)	429(93.7,91.0–95.7%)	414 (92.0,89.1–94.3%)

*Possible failure: lost to follow-up or withdrawal (a subject** who received high dose of treatment or subjects antibiotics with anti-malarial activity or malaria smear results invalidated after quality control) or subjects for which filters paper were not analyzed or were undetermined result by PCR

**One subject received on day 0 one dose of a junior tablet of AS-SMP instead of a baby tablet as described in the study procedures and treatment. There was no adverse event found to be related to that misuse of the study drug.

**Table 3:** Efficacy evaluation on Day 28 after PCR correction* Per-Protocol Analysis

**Efficacy evaluation**	AS-SMP 3 days (N = 435)	AS-SMP 24 hours (N = 418)	AL 3 days (N = 392)
ETF – n (%)	0	0	1(0.3%)
LCF – n (%, 95%CI)	3 (0.7%,0.1–2.0)	2 (0.5%,0.1–1.7)	0
LPF – n (%)	0	0	0
ACPR – n (%, 95%CI)	432 (99.3%,98.0–99.9)	416 (99.5%,98.3–99.9)	391 (99.7%,98.6–100.0)
Reinfection rate – (%, 95%CI)	12 (2.8%,1.4–4.8)	13 (3.1%,1.7–5.3)	14 (3.6%,2.0–5.9)

*Subjects for which filters paper were not analyzed or were undetermined result by PCR or protocol violation cases were excluded

The PCR uncorrected 28-day cure rates were similar among treatment groups: 93.7%(446/476) in the AS-SMP three-day group, 91.1%(417/458) in the AS-SMP 24-hour group and 89.6%(403/450) in the AL group in the ITT population. Likewise, the PCR uncorrected 28-day cure rates were similar among treatment groups: 95.9% (422/440) in the AS-SMP three-day group, 96.0%(404/421) in the AS-SMP 24-hour group and 96.0%(380/396) in the AL group in the PP population. The reinfection rates were similar among treatment groups: 2.8% in the AS-SMP three-day group, 3.1% in the AS-SMP 24-hour group and 3.6% in the AL group in the PP population (Table 3).

Overall, there was no discernable difference between study centers for the primary efficacy variable (Table 4). There was also no discernable difference in efficacy between the < 5 years of age group and greater or equal to 5 years of age group (Table 5).

**Table 4:** Efficacy on D28 after PCR correction by centre (*Per-Protocol Analysis*)

**Efficacy evaluation**	AS-SMP 3 days (N = 435)	AS-SMP 24 hours (N = 418)	AL 3 days (N = 392)
**Cameroon (n)**	85	75	70
ACPR – n (%, 95%CI)	84 (98.8%,93.6–100.0)	74 (98.7,92.8–100.0%)	69 (98.6%,92.3–100.0)
**Mali (n)**	86	82	85
ACPR – n(%, 95%CI)	86 (100%,95.8–100.0)	82 (100%,95.6–100.0)	85 (100%,95.7–100.0)
**Sudan (n)**	84	92	73
ACPR – n(%, 95%CI)	84 (100%,95.7–100.0)	92 (100%,96.1–100.0)	73 (100%,95.1–100.0)
**Rwanda (n)**	180	169	164
ACPR – n(%, 95%CI)	178 (98.9%,96.0–99.9)	168 (99.4%,96.7–100.0)	164 (100%,97.8–100.0)

*Subjects for which filters paper were not analyzed or were undetermined result by PCR or protocol violation cases were excluded

**Table 5:** Efficacy on D28 after PCR correction by age category (*Per-Protocol Analysis*)

**Efficacy evaluation**	AS-SMP 3 days	AS-SMP 24 hours	AL 3 days	P
**Age < 5 years – n**	144	111	126	
ACPR – n (%,95%CI)	144 (100%,97.5–100.0)	111 (100%,96.7–100.0)	126 (100%,97.1–100.0)	--
**Age >= 5 years – n**	293	308	268	
ACPR – n (%,95%CI)	290 (99.0%,97.0–99.8)	306 (99.4%,97.7–99.9)	267 (99.6%,97.9–100.0)	0.78 (Fisher)

*Subjects for which filters paper were not analyzed or were undetermined result by PCR or protocol violation cases were excluded

**Figure 2:**
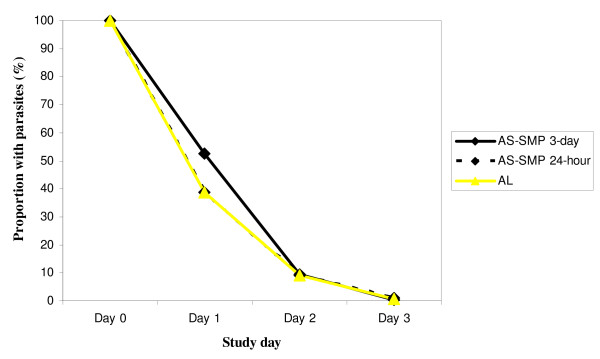
**Proportion of participants with parasite during the first 3 days after treatment**.

There was a rapid parasite clearance in all treatment groups (Figure 2): 47.5%(206/434) in the AS-SMP three-day group, 61.2% (255/417) in the AS-SMP 24-hour group and 51.8%(203/392) in AL group cleared parasite on Day 1; p < 0.001. On Day 2, 90.5%(391/432) in the AS-SMP three-day group, 91.0% (376/413) in the AS-SMP 24-hour group and 90.8%(357/393) in AL group cleared parasite = 0.96. On Day 3, 99.5%(433/435) in the AS-SMP three-day group, 99.0% (410/414) in the AS-SMP 24-hour group and 99.2%(388/391) in AL group cleared parasite = 0.68.

For malaria fever clearance: only 2/405 patients (0.3%) and 2/387 (0.3%) in the AS-SMP 24-hour group and in the AL group on day 2 had fever and parasite while the fever clearance was total in all treatment groups on day 3. Gametocyte carriage decreased from baseline to day 28. At baseline on Day 0, 6/432 patients (1.4%) in the AS-SMP three-day group, 8/394 (2.0%) and 16/418 (3.8%) in the AS-SMP 24-hour group were carrying gametocytes. Only 2/427 patients (0.5%) in the AS-SMP 3 group, 0/387 (0%) and 10/410 (2.4%) in the AS-SMP 24-hour group were carrying gametocytes on day 7; p = 0.001.

There was one patient with ETF in the AL group. Few patients developed severe malaria: four patients in the AL group and two in the AS-SMP 24-hour group, all treated successfully with other anti-malarias treatment (by injection).

### Tolerability and safety

The adverse events probably related to study drug were gastrointestinal signs/symptoms (vomiting, nausea, diarrhoea, abdominal pain, anorexia), dizziness, rash and weakness. Gastrointestinal signs/symptoms, such as vomiting and diarrhoea, were slightly higher in AS-SMP 24-hour group: for vomiting, it was 7.0% (n = 458), 4.6% (n = 476) and 2.2% (n = 450) for AS-SMP 24-hour group, AS-SMP three-day group and AL group respectively; p = 0.003. For diarrhoea it was 3.3% (n = 458), 0.6% (n = 476) and 1.3% (n = 450) for AS-SMP 24-hour group, AS-SMP three-day group and AL group respectively; p = 0.006 (Table 6). There was no death reported. There were few serious adverse events reported (AL, 5/450 [1.1%]; AS-SMP 24 hours, 2/458 [0.4%]), all being severe malaria except for one severe case of anaemia in AL group, which occurred a few days after study drug intake. No clinically significant laboratory abnormal value (haemogram or liver enzymes, such as ALT or creatinine or bilirubine) related to study dug has been reported in any treatment group.

**Table 6:** The frequency of adverse events mild to moderate grades from day 1 to day 7 (related or not) after treatment initiation

	AS-SMP 3 days (A) (N = 476)	AS-SMP 24 hours (B) (N = 458)	AL 3 days (C) (N = 450)	P-value
**Vomiting – n (%)**	22 (4.6)	32 (7.0)	10 (2.2)	0.003
**Nausea-n (%)**	15 (3.2)	19 (4.2)	14 (3.1)	0.6
**Headache – n (%)**	5 (1.1)	10 (2.2)	7 (1.6)	0.4
**Weakness – n (%)**	11 (2.3)	5 (1.1)	2 (0.4)	0.04
**Anorexia – n (%)**	16 (3.4)	11 (2.4)	17 (3.8)	0.5
**Dizziness – n (%)**	13 (2.7)	14 (3.1)	10 (2.2)	0.7
**Diarrhea – n (%)**	3 (0.6)	15 (3.3)	6 (1.3)	0.006
**Rash – n (%)**	3 (0.6)	5 (1.1)	1 (0.2)	0.3
**Abdominal pain – n (%)**	21 (4.4)	21 (4.6)	17 (3.8)	0.8
Others* – n (%)	15 (3.2)	10 (2.2)	12 (2.7)	0.7
**Total – n (%)**	124 (26.1)	142 (31.0)	96 (21.3)	0.004

Data are no. (%) of participants with the sign or symptom from day 1 to day 7 after treatment

* Others are: infections (skin, respiratory, intestinal...) pains...

## Discussion

Malaria remains a major health problem characterized by high mortality and serious morbidity in particular with children less than five years of age [1-3]. Malaria also impacts on the development of children and later on the economy of the country [24], so that adequate treatment compliance may play key role in lowering the malaria burden. With the combination of artesunate-sulphadoxine-pyrimethamine (AS-SP) it is impossible to make a FDC tablet, since the total dose of SP must be swallowed at once on the first day while the artesunate dose is spread over three days. With other forms of ACT, multiple tablets may be administered daily over three days [25]. With the dihydroartemisinin-piperaquine combination, treatment is over three days and, although an interesting combination [26], the product suffers from the chemical instability of DHA (considerable breakdown of DHA when exposed to higher temperatures) [27,28]. With the AL combination (Coartem^®^) problems such as two doses per day over three days and high re-infection rate in areas with high transmission intensity are a disadvantage [26]. The options for the next ten years are limited and, therefore, new anti-malarials are needed [29].

The current study brings interesting information regarding a new ACT. Previous studies had shown AS-SMP to be adequate [10-13] and the current study confirmed this on a larger number of patients from four different geographical areas of Africa.

The study shows that the efficacy of the AS-SMP 24-hour or AS-SMP three-day fixed dose treatment in patients with acute uncomplicated falciparum malaria was not inferior to AL, and had a comparable safety profile except for vomiting slightly higher in the AS-SMP 24-hour group.

PCR-corrected 28-day cure rates were high in both ITT and PP analysis in the different treatment groups. The efficacy rate was comparable across the countries, although the study was not powered enough for comparing the three treatment groups within country. Moreover, there was no difference among the three treatment groups in term of clearance of fever or parasite (Figure 2), which was rapid for all treatments. The percentage of excluded patients (9.7%) was below the pre-specified value of 15%. Results from ITT and PP analyses were in good agreement.

The PCR-corrected 28-day cure rates in the AS-SMP 24-hour or AS-SMP three-day group were high and similar to findings in previously published investigations on the efficacy of co-blister AS-SMP three-day or fixed AS-SMP [10-13]. Although similar reinfection rates have been found in this study (Table 3) a longer period of follow up would probably have brought out bigger differences in recurrence rates between treatments.

The AS-SMP was well tolerated in general, particularly the AS-SMP three-day regimen. No new safety signal was detected and observations were in line with previous findings [12,13]. Most commonly reported AEs were typical symptoms of malaria. The most frequently experienced drug-related AE was vomiting, but this is also typical for malaria in the first days. Overall, the safety data gathered in this study did not support any increased risk for AEs with AS-SMP 24-hour regimen except for vomiting.

This study was performed under supervised study conditions and may, therefore, not entirely mirror normal outpatient practice, which could be considered as a limitation of the present trial. To improve the compliance, a fixed dose of AS-SMP three-day treatment (once daily) or moreover AS-SMP 24-hour treatment (0–12 h-24 h) has been developed. This study shows that AS-SMP three-day or AS-SMP 24 hours is as efficacious as the AL three-day treatment (two doses a day).

In this study, it was clearly demonstrated that the shortening of the dose interval between tablets from three days to one day (24 hours) did not compromise the outcome of the malaria course. However, the second dose of the 12-hour interval administration drug of AS-SMP and second dose administration drug of AL may fall outside the regular working hours of the health centers and also may fall to patients' bed time and therefore may impact on treatment compliance. In term of efficacy, there was no difference between treatment groups and the result in each treatment group approaches 100% in the PP analysis (ACPR = 99%). This is rather remarkable since the study was carried out in areas where resistance to SP is high. This holds for Rwanda where the SP resistance is estimated to exceed 25% [18]. Also in Cameroun the relative resistance exceeded 10% [14,15]. In other areas, resistance to SP is estimated to be ranging from 5% to 30% (Sudan) [19,20]. Recent publications drew attention to the suitability of the use of ACT in an area where resistance to the partner drug is high and argued that in areas with high resistance to pyrimethamine alone or combined with sulphadoxine, the combination of SP with AS might not be a valid alternative therapy [30]. However, in spite of the high SP resistance in Kigali, Rwanda, the combination with AS gave a ACPR rate of 90.3% [11]. In the same study, the combination of AS/SMP (Co-arinate^® ^co-blister) gave a 96.6% ACPR. The high cure rate of AS/SMP compared to AS/SP in the Rwanda study is also confirmed in this multicentre study; confirming the chemical activity differences between SMP and SP [10] even though the pyrimethamine component is similar for both drugs. Additional file (additional file 1) has been provided in order to clarify in more details the difference between sulfamethoxypyrazine and sulphadoxine. Also the possibility to combine AS with a drug showing a relative high resistance, such as SP, was confirmed in a recent study in Benin and Ghana [31,32]. This should not be considered as surprising since, in Thailand in the nineties, the combination of AS with mefloquine gave excellent results in spite of a high resistance to mefloquine [33]. The study from Rwanda and Benin using AS+SP are emphasizing once more that a relatively high resistance to one of the longer acting partner drugs may not necessarily preclude it from being used in an ACT.

## Conclusion

AS-SMP three days or AS-SMP 24 hours are safe, are as efficacious as AL, and are well tolerated, although vomiting and diarrhoea were slightly higher in the AS-SMP 24-hour group than other groups.

## Competing interests

The authors declare that they have no competing interests except for FHJ who is an employee of Dafra Pharma. No other author received any honoraria or salary from Dafra Pharma.

## Authors' contributions

All authors contributed to the design of the study and assisted with data interpretation. IS, WM, RS and IA coordinated the study and supervised the enrollment and follow-up of patients. FH J, AD, OKD, and AD participated in study design. IS and AD contributed in data management and analysis. AD did the molecular analysis in determining the status of recrudescence or reinfection. KS, HM, OBT, ND and YTD collected the data. All authors participated in the preparation of the manuscript and approved the final version.

## Supplementary Material

Additional file 1 **Sulfamethoxypyrazine-Pyrimethamine *v.s *Sulfadoxine-Pyrimethamine**. History and comparison of Sulfamethoxypyrazine-Pyrimethamine_vs_Sulfadoxine-Pyrimethamine Click here for file
