# Supplementary material for: Efficacy and safety of a fixed dose artesunate-sulphamethoxypyrazine-pyrimethamine compared to artemether-lumefantrine for the treatment of uncomplicated falciparum malaria across Africa: a randomized multi-centre trial
Source: Malar J. 2009 Apr 14;8:63. doi: 10.1186/1475-2875-8-63 (PMC2678145; doi:10.1186/1475-2875-8-63)
Supplement: Additional file 1 — Sulfamethoxypyrazine-Pyrimethamine v.s Sulfadoxine-Pyrimethamine. History and comparison of Sulfamethoxypyrazine-Pyrimethamine_vs_Sulfadoxine-Pyrimethamine [file 1475-2875-8-63-S1.doc]

Sulfamethoxypyrazine (SMP) or sulfalene is the last of the long acting sulfonamides synthetised. Its structure was the result of profound structure activity work of the mid sixties. The designers had aimed for a long acting sulphonamide with a rather long elimination half life and a limited plasma protein binding. The structure that finally was proposed and synthetised had a pyrazine ring substitution on the N1 amide function and in this ring a methoxy-group was planted in ortho position. The Italian company Farmitalia made the compound and developed it as a drug. As a sulphonamide it was rather revolutionary because of its low dosing. The work around this drug formed the basis of pharmacokinetic work and led to the development of clinical pharmacology. SMP has an elimination half life of 65-80 hours and a protein binding of only 65%. This means that permanently a high non protein bound fraction is available for penetration and distribution in the plasma water and in interstitial fluids. The drug penetrates cerebral meninges and can most successfully be used for treatment of bacterial meningitis. The drug is metabolised by acetylation of the primary amino group (N4) which has an excellent solubility in urine. Although the introduction of the drug was successful it had the massive introduction of antibiotics against and cheap sulfonamides were no match against expensive antibiotics. When Hofman la Roche introduced sulfadoxine (a long acting sulphonamide not introduced into western medicine) in combination with pyrimethamine, sulfadoxine-pyrimethamine under the brand name Fansidar®, an equivalent drug with sulfalene followed being Metakelfin®. Marketing efforts of Roche were much stronger and hence the popularity of Fansidar® dominated Metakelfin®. The latter, however, was recognised as the better combination by WHO and Molyneux used it massively in a malaria eradication programme in Wewstern Sudan. Its results were published in the Garki study report (ref). A remarkable feature of sulfalene is its nearly absolute safety. Repeated screening of all publications since 1965 (using pubmed) did not bring forward any negative effect that can be related to the sulphonamide. In the seventies and eighties sulfalene was still quite popular in eastern European medicine. The drug is now owned by Pfizer and the European registration in Italy is still being maintained.
